# Could caregiver reporting adherence help detect virological failure in Cameroonian early treated HIV-infected infants?

**DOI:** 10.1186/s12887-015-0451-3

**Published:** 2015-09-21

**Authors:** Francis Ateba Ndongo, Josiane Warszawski, Gaetan Texier, Ida Penda, Suzie Tetang Ndiang, Jean-Audrey Ndongo, Georgette Guemkam, Casimir Ledoux Sofeu, Anfumbom Kfutwah, Albert Faye, Philippe Msellati, Mathurin Cyrille Tejiokem, the ANRS-PEDIACAM study group

**Affiliations:** Université Paris-Sud; Centre Mère et Enfant de la Fondation Chantal Biya, POB 1936, Yaounde, Cameroon; Université Paris-Sud; Assistance Publique des Hôpitaux de Paris, CESP INSERM U1018, team 4 “HIV and STD”; Hôpital Bicêtre, 94276 Le Kremlin-Bicêtre, France; SESSTIM (UMR 912) Aix-Marseille Université, Marseille, France; Centre Pasteur du Cameroun, Service d’Epidémiologie et de Santé Publique, POB 1274, Yaounde, Cameroon; Université Douala; Hôpital Laquintinie, POB 4035 Douala, Cameroon; Centre Hospitalier d’Essos, POB 5777, Yaounde, Cameroon; Centre Mère et Enfant de la Fondation Chantal Biya, POB 1936, Yaounde, Cameroon; Université Yaoundé I; Centre Pasteur du Cameroun, Service d’Epidémiologie et de Santé Publique, POB 1274, Yaounde, Cameroon; Centre Pasteur du Cameroun, Service de Virologie, POB 1274, Yaounde, Cameroon; Université Paris Diderot, Sorbonne Paris Cité; Assistance Publique des Hôpitaux de Paris, Pédiatrie Générale, Hôpital Robert Debré, Paris, France; UMI 233, IRD, Université Montpellier, POB 64501, 34394 Montpellier, France

**Keywords:** Antiretroviral therapy, Infants, Adherence reporting, Virological failure, Resource-limited settings

## Abstract

**Background:**

Viral load is still the marker of choice for monitoring adherence to combined antiretroviral therapy (cART) and confirming the success of HIV treatment. Unfortunately it is difficult to access in many resource-poor settings. We aimed to measure the performance of caregiver reporting adherence for detecting virological failure in routine practice during the first 2 years after cART initiation in infants.

**Methods:**

PEDIACAM is an ongoing prospective cohort study including HIV1-infected infants diagnosed before 7 months of age between November 2007 and October 2011 in Cameroon. Adherence was assessed using a questionnaire administered every 3 months from cART initiation; the HIV-RNA viral load was determined at the same visits. Virological failure was defined as having a viral load ≥ 1000 cp/mL at 3 and 12 months after cART initiation or having a viral load ≥ 400 cp/mL at 24 months after cART initiation. The performance of each current missed and cumulative missed dose defined according to adherence as reported by caregiver was assessed using the viral load as the gold standard.

**Results:**

cART was initiated at a median age of 4 months (IQR: 3–6) in the 167 infants included. The cumulative missed dose showed the best overall performance for detecting virological failure after 12 months of cART (AUC test, *p* = 0.005, LR + =4.4 and LR− = 0.4). Whatever the adherence reporting criterion, the negative predictive value was high (NPV ≥ 75 %) 12 and 24 months after cART initiation, whereas the positive predictive value was low (PPV ≤ 50 %).

**Conclusions:**

The adherence questionnaire administered by the health care provider to the infants’ caregivers is not reliable for detecting virological failure in routine practice: its positive predictive value is low. However, the cumulative missed dose measurement may be a reliable predictor of virological success, particularly after 12 months of cART, given its high negative predictive value.

## Background

Mortality and morbidity due to HIV/AIDS in both adults and children has been dramatically reduced by combined antiretroviral therapy (cART) [1–3]. The CHER study showed that early initiation of cART in infants, before 3 months of age, reduced mortality by around 75 % [1]. Thus, in 2008 the WHO recommended that all HIV-infected infants diagnosed before age 2 years be immediately treated, regardless of clinical, immune or virological status; in 2013, the WHO further recommended a rapid scale-up of systematic cART to any HIV-infected child under 5 years of age [1–4].

Satisfactory virological and clinical responses require adherence to cART; adherence also limits the emergence of antiretroviral drug resistance, particularly important in resource-limited settings with restricted cART regimen options [5–11]. Viral load measurement is the best marker of response to cART which is strongly reflective of adherence to cART in patients whose strains are susceptible to antiretroviral treatment they are receiving*.* But it is difficult to access for routine use in many resource-limited settings. There are other indicators some of which have been used in biomedical research: direct measures, including assaying plasma for drugs or drug metabolites; and indirect evaluations including self-reports, electronic drug monitoring, pill counts and pharmacy refill records [12–18]. Electronic drug monitoring appears to be the most sensitive indirect method for detecting missed doses of medication, but is difficult to implement in a resource-limited setting [13].

Pediatric cART is increasingly widely used in low-income countries and therefore there is a corresponding need for reliable methods, easier to access than viral load determinations, for assessing infant adherence to cART in routine practice. We are unaware of any previous study in Sub-Saharan Africa using indirect evaluations specifically to evaluate the adherence to cART among infants.

The main objective of this study was to assess the performance of caregiver adherence reporting questionnaires for detecting virological failure in routine practice during the first 2 years of cART in infants in Cameroon.

## Methods

### The ANRS-12140 Pediacam Study

PEDIACAM is a prospective cohort study of HIV-infected infants included between November 2007 and October 2011 in three referral hospitals in Cameroon: the Centre Mère et Enfant de la Fondation Chantal Biya (CME/FCB) and Centre Hospitalier d’Essos (CHE) both in Yaounde and Hôpital Laquintinie de Douala (HLD) in Douala. The inclusions in PEDIACAM were organized in two phases and are described elsewhere [18]. In brief, during the first phase, infants born to HIV-infected mothers and those born to HIV-uninfected mothers were matched according to gender and site of recruitment during the first week of life and followed until the fourteenth week. During the follow-up period, all infants received routine vaccines according to the Expanded Program of Immunization, planned at 6, 10 and 14 weeks. HIV-exposed infants underwent HIV molecular diagnostic tests (HIV-DNA PCR or HIV-RNA PCR) at 6 weeks of age and the results were available at week 10. HIV-positive infants were confirmed by testing a second sample collected at age 10 weeks. Breastfed infants with previously negative HIV tests were retested 6 weeks after weaning. All HIV-infected infants and subsamples of uninfected HIV-exposed infants and HIV-negative unexposed infants were eligible for the second phase of follow-up planned to continue to age 2 years. Inclusion into phase 2 was also offered directly to HIV-infected infants not followed from the first week of life but diagnosed before the age of 7 months.

Overall, 210 HIV-infected infants were included in the second phase: cART was proposed systematically to these children as soon as the HIV status was confirmed. The initial cART regimen depended on PMTCT (Prevention of Mother-to-Child Transmission) history and followed the Cameroonian guidelines at the time of the study which recommended zidovudine (or abacavir or stavudine if anemic), lamivudine and lopinavir/r if there was any use of nevirapine for PMTCT (or nevirapine otherwise). The caregivers were asked to administer the drugs to the infants twice daily (every 12 h). A standardized questionnaire was administered every 3 months after initiation of cART by a health care provider (physician, nurse, psychosocial worker or pharmacist) to assess infant adherence to cART during the period and to identify and help families with any difficulties. In total, eight questionnaires were planned from month 3 (M3) to month 24 (M24) of follow-up. The HIV viral load was measured at the same follow-up time points using RT PCR (Biocentric, Bandol, France).

### Caregiver adherence questionnaire

The adherence questionnaire was similar to the questionnaire used in the PENTA trial, with some adaptations [16]. It included questions about: the relationship between the caregiver and the child; difficulties experienced during drug administration; the most difficult dose to remember; how administration of cART interfered with everyday life; reasons for the difficulties identified; and the caregiver’s recall of missed doses in the past 3 and 14 days.

### Study population

**Fig. 1:**
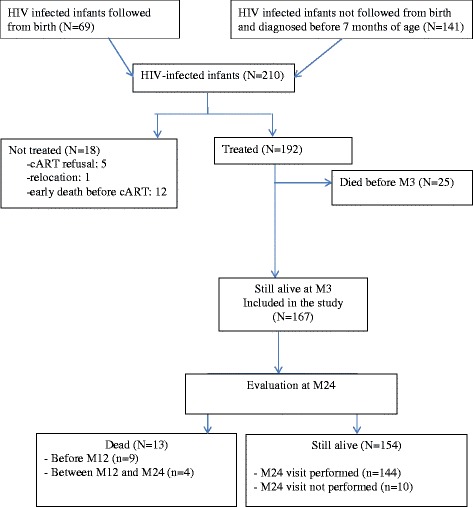
Cohort description (ANRS-PEDIACAM Study, 2008-2013, Cameroon). *Legend:* N, number; cART, combined antiretroviral therapy; Mx, delay from ART initiation to the current visit, e.g. «M3» means «3 months after cART initiation»

Among the 210 HIV-infected infants included in phase 2 of the PEDIACAM study, 13 died before treatment started and five refused cART. For the 192 infants who initiated cART, 25 died during the first three months of treatment. Thus, 167 HIV-infected infants treated for at least three months were included in this analysis (Fig. 1). Infants without virological data at a particular time point (because of death or missing values) were not included in the analysis at that particular time point. There were: 33 (19.8 %) such children at M3, 9 (5.4 %) at M12 and 6 (3.6 %) at M24.

### Variables and statistical analysis

The quarterly monitoring values used were the nearest measurement within a ±1.5 month interval of each of the following times after cART initiation: M3, M6, M9, M12, M15, M18, M21 and M24.

The main outcome was virological failure at M3, M12 and M24, defined as viral load higher than 1000 copies/mL (at M3 or M12) or higher than 400 copies/mL (at M24). We chose a higher threshold for M3 and M12 because a previous African study of HIV-infected infants under 12 months of age reported very high viral loads at baseline and that treatment duration contributed to lower viral loads at M24 [19]. HIV resistance genotyping by sequence amplification and interpretation according to the French ANRS interpretation algorithms was carried out for selected cases of virological failure associated with reporting suggesting adequate adherence.

The outcome measures also included non-adherence at the various time points recorded as: “current missed doses” (criterion A) defined as the number of cART doses not administered to an infant at a particular time point (M3, M12 or M24) and “cumulative missed doses” (criterion B) which was the sum of cART doses reported as not administered every three months during the corresponding interval M3 to M12 or M3 to M24. We also used mixed criteria (A or B, A and B). When a quarterly visit was lacking, all the cART doses for this visit were considered to be missing. When a quarterly visit was performed but the adherence questionnaire not completed, we considered the previous number of missed doses for that particular visit.

Many different thresholds (5, 10, 15 and 20 %) for current missed doses have been used in previous studies in the literature to define non-adherent HIV-infected patients [13, 14, 16, 19–21]. We compared many of these thresholds, using receiver operating characteristic (ROC) curves, maximizing the sensitivity, to determine cutoff points for missed dose values that are optimal for detecting virological failure at the various time points (viral load ≥1000 copies/mL at M3 and M12, or ≥400 copies/mL at M24). Infants with values above this cut-off were defined as non-adherent. We performed area under the curve (AUC) tests for criteria A and B for M3, M12 and M24. We estimated the sensitivity (proportion identified as non-adherent among children with virological failure) and specificity (proportion identified as adherent among those with virological success) of the approach. We also estimated positive predictive value (PPV; probability of virological failure among children whose caregiver reported non adherence) and negative predictive value (NPV; probability of virological success among children whose caregiver reported adherence). We then estimated positive and negative likelihood ratios (LR): the ratio of probability to be “non-adherent” (LR+) or to be “adherent” (LR−) for a child with virological failure versus virological success.

We studied associations between missed dose A or B criteria and various other items in the adherence questionnaire: problems with giving medication (difficulties during drug administration, the most difficult dose to remember, how administration of cART interferes with everyday life, reasons for the difficulties), type of caregiver and health care provider, the time point of the visit. Univariate analysis was first performed, using Chi square or Fisher’s exact test, as appropriate. Adjusted odds ratios (aOR) were estimated in multivariate logistic regression models, with reported missed dose as the dependent variable. For all analyses, p-values less than 0.05 (two-sided) were considered statistically significant. All the statistical analyses were performed using STATA 12.0 (StataCorp, College Station, TX).

### Ethical consideration

The ANRS-PEDIACAM study was granted ethical approval in Cameroon by the National Ethics Committee and in France by the Biomedical Research Committee of the Pasteur Institute of Paris. The Cameroon Ministry of Public Health gave administrative authorization to start the study. Caregivers signed written informed consent for participating in the study.

## Results

### Study population

Among the 167 HIV-infected children included (Fig. 1) in this analysis, 45.5 % were boys. Half of the infants were living with both of the parents, 38 % with their mothers only and 12 % with other caregivers; 6.6 % were motherless orphans (Table 1). cART was initiated at median age of 4 months (IQR: 3–9), when the median viral load (VL) was 6.5 log10 copies/mL (IQR: 6.0–6.9) and median CD4 percentage 22 % (IQR: 15–31 %). Respectively 34 and 64 % of infants started cART with nevirapine-based and lopinavir-based regimens*.* Three quarters of the infants were enrolled in Yaounde. Two years after cART initiation, 92.2 % (154/167) of infants were alive, and 144 attended the M24 visit. Thirteen infants died, including nine before M12, and four between M12 and M24 (Table 2).

**Table 1:** Description of baseline characteristics of HIV-infected infants treated by cART for at least 3 months (ANRS-PEDIACAM Study, 2008-2013, Cameroon)

	*N* = 167	
Variables (n, % of missing data)	*N*	% (n) or median (IQR)
Male sex	167	45.5 (76)
Infant group at inclusion	167	
Followed since birth		37.7 (63)
Not followed since birth but diagnosed before age 7 months		62.3 (130)
Recruitment sites	167	
CME/FCB		47.3 (79)
HLD		23.4 (39)
CHE		29.3 (49)
History of breastfeeding	110	51.8 (57)
Vital status of parents at cART initiation	167	
Mother dead		6.6 (11)
Father dead		3.6 (6)
Vital status of the father unknown (or data missing)		6.6 (11)
Infants living with:	167	
Both parents		50.3 (84)
Mother only		37.7 (63)
Other caregivers		12.0 (20)
Presence of functional fridge at home	115	43.5 (50)
Difficulty with remembering to give cART medication		
M3	138	33.3 (46)
M12	123	24.4 (30)
M24	81	29.6 (24)
Clinical status at cART initiation	167	
WAZ (median, IQR)		−0.2 (−0.4; −0.1)
History of hospitalization		41.3 (69)
CD4 count (%) at cART initiation (median, IQR)	161	23 (15 ; 32)
Viral load at cART initiation (log10 cp/mL) (median, IQR)	166	6.5 (6.0 ; 6.9)
Age at cART initiation	167	
< 6 months		80.8 (135)
6–12 months		16.8 (28)
> 12 months		2.4 (4)
In months (median, IQR)		4.0 (3.0–9.0)
Type of first cART	167	
Protease inhibitor-based regimen		*63.9 (110)*
Nevirapine-based regimen		*34.1 (57)*

*N* total number, *n* number of children in the group, *CME/FCB* Centre Mère et Enfant/Fondation Chantal Biya, Yaounde, *HLD* Hôpital Laquintinie, Douala, *CHE* Centre Hospitalier d’Essos, Yaounde, *Mx* delay from cART initiation to the current visit, e.g. «M3» means «3 months after cART initiation», *cART* combination antiretroviral therapy, *%* percentage, *n* number of patients in the cell, *WAZ* Weight for Age Z score based on WHO reference tables, *IQR* interquartile range, *CD4 lymphocytes that have CD4 marker*

**Table 2:** Follow-up of infants treated by cART for at least 3 months (ANRS-PEDIACAM Study, 2008–2013, Cameroon)

	*N* = 167		
	M3 visit	M12 visit	M24 visit
	% (*n*)	% (*n*)	% (*n*)
Dead	0.0 (0)	5.3 (9)^a^	2.4 (4)^b^
Visit not performed	20.4 (34)	23.4 (39)	29.1 (64)
Visit performed	79.6 (133)	71.3 (119)	48.5 (81)
Viral load (cp/mL)			
< 400	35.3 (59)	52.7 (88)	34.1 (57)
[400-1000]	15.6 (26)	4.8 (8)	3.6 (6)
≥ 1000	36.5 (61)	18.0 (30)	9.6 (16)
not measured	12.6 (18)	24.5 (41)	59.3 (88)
Accompanied by:			
Mother	79.0 (105)	73.1 (87)	71.6 (58)
*Grandmother*	8.3 (11)	8.4 (10)	8.6 (7)
Aunt	5.9 (8)	5.9 (7)	5.0 (4)
Other	6.8 (9)	12.6 (15)	14.8 (12)
Questionnaire completed	*N* = 151	*N* = 126	*N* = 126
Questionnaire completed and viral load measured	*N* = 144	*N* = 125	*N* = 67

*cART* combined antiretroviral therapy, *N* total number, *Mx* delay from ART initiation to the current visit, e.g. «M3» means «3 months after cART initiation», *%* percentage; *n* number of patients in the cell

^a^<12 months

^b^12 to 24 months

### Adherence reporting by caregivers

Adherence questionnaires were administered by physicians (34.8 %), pharmacists (31.5 %), psychosocial workers (19.8 %) and nurses (13.9 %), and the respondent was mostly the mother (in 79.0 % of cases at M3, 73.1 % at M12 and 71.6 % at M24). According to respondents, cART medication was usually administered by the mother (84.3 % at M3, 81.1 % at M12 and 77.9 % at M24) although other caregivers may also have been involved in cART administration (63.8, 70.2 and 73.8 %, respectively).

For current missed doses (criterion A), the cutoff point which provided the best compromise between sensitivity and specificity, according ROC curves, was ≥1 missed dose at all visits (Table 3). For cumulative missed doses (criterion B), the best cutoff points were ≥2 missed doses at M12 and ≥8 missed doses at M24. With these cutoff points, the proportions of non-adherent children according to reported current missed dose were 27.4 % (40/146) at M3, 11.1 % (14/126) at M12 and 13.9 % (11/79) at M24. The proportions, based on the cutoff values for cumulative missed doses, were 23.8 % (30/126) at M12 and 62.0 % (49/79) at M24.

**Table 3:** Performance of reported adherence for detecting virological failure in HIV-infected infants treated early by cART for at least 3 months (ANRS-PEDIACAM Study, 2008–2013, Cameroon)

3a - All children				AUC		Sensitivity		Specificity		PPV		NPV		LR+	LR−
	N	%	n	%	p	%	(n/N)	%	(n/N)	%	(n/N)	%	(n/N)		
At M3	146														
VL ≥ 1000 cp /mL		41.8	(61)												
≥ 1 current missed (A)		27. 4	(40)	49.4	0.870	26.2	(16/61)	71.8	(61/85)	40.0	(16/40)	57.5	(61/106)	1.0	1.0
At M12	126														
VL ≥ 1000 cp /mL		23.8	(30)												
≥ 1 current missed (A)		11.1	(14)	53.8	0.260	16.7	(5/30)	90.6	(87/96)	35.7	(5/14)	77.7	(87/112)	1.8	0.9
≥ 2 cumulative missed (B)		23.8	(30)	64.2	0.005	63.3	(19/30)	67.7	(65/96)	38.0	(19/50)	85.5	(65/76)	*2.0*	*0.5*
A or B						63.3	(19/30)	65.6	(63/96)	36.5	(19/52)	85.1	(63/74)	1.8	0.6
A and B						16.7	(5/30)	89.6	(86/96)	41.7	(5/12)	78.1	(89/114)	2.3	1.0
At M24	79														
VL > 400 cp /mL		27.8	(22)												
≥ 1 current missed (A)		13.9	(11)	56.6	0.100	22.7	(5/22)	89.5	(51/57)	45.5	(5/11)	75.0	(51/68)	2.2	0.9
≥ 8 cumulative missed (B)		62.0	(49)	57.7	0.130	72.7	(16/22)	42.1	(24/57)	32.7	(16/49)	80.0	(24/30)	1.3	0.7
A or B						72.7	(16/22)	40.4	(23/57)	32.0	(16/50)	79.3	(23/29)	1.2	0.7
A and B						22.7	(5/22)	91.2	(52/57)	50.0	(5/10)	75.4	(52/69)	2.6	0.9
3b Children accompanied by mothers				AUC		Sensitivity		Specificity		PPV		NPV		LR+	LR−
	N	%	n	%	p	%	(n/N)	%	(n/N)	%	(n/N)	%	(n/N)		
At M3	116														
VL ≥ 1000 cp /mL		41.4	(48)												
≥ 1 current missed (A)		25.0	(29)	49.9	0.510	21.7	(12/48)	72.6	(51/68)	41.4	(12/29)	58.6	(51/87)	1.0	1.0
At M12															
VL ≥ 1000 cp /mL	92	22.8	(21)												
≥ 1 current missed (A)	92	13.0	(13)	50.7	0.120	14.3	(3/21)	87.3	(62/71)	25.0	(3/12)	77.5	(62/80)	1.1	1.0
≥2 cumulative missed (B)	67	30.4	(21)	68.1	0.010	64.3	(9/14)	77.4	(43/55)	42.9	(9/21)	89.6	(43/48)	3.0	0.5
A or B	92					76.2	16/21)	57.8	(41/71)	34.8	(16/46)	89.1	(41/46)	1.8	0.4
A and B	92					14.3	(3/21)	95.8	(68/71)	50.0	(3/6)	79.1	(68/86)	3.4	0.9
At M24															
VL > 400 cp /mL	53	24.5	(13)												
≥ 1 current missed (A)	53	9.4	(5)	51.4	0.410	7.7	(1/13)	90.0	(36/40)	20.0	(1/5)	75.0	(36/48)	0.8	1.0
≥ 8 cumulative missed (B)	4	0	(0)	NA	NA	NA	NA	NA	NA	NA	NA	NA	NA	NA	NA
A or B	53					100	(13/13)	5.0	(2/40)	25.5	13/51	100	(2/2)	1.1	0.0
A and B	4					NA	NA	NA	NA	NA	NA	NA	NA	NA	NA

*cART* combined antiretroviral therapy, *VL* viral load, *Mx* duration covered from cART initiation to current visit, e.g. «M3» means «3 months duration since cART initiation», *N* total number, *%* percentage, *n* number of patients in the group, *IQR* interquartile range, *current* number of missed doses during the previous 3 days, *cumulative* total number of accumulated missed doses from M3 to current visit (M12 or M24), *N* total size, *PPV* positive predictive value, *NPV* negative predictive value, *p* AUC test, *LR*+ positive likelihood ratio, *LR−* negative likelihood ratio, *NA* not applicable, due to absence of patients with both virological failure and non-adherence

**Fig. 2:**
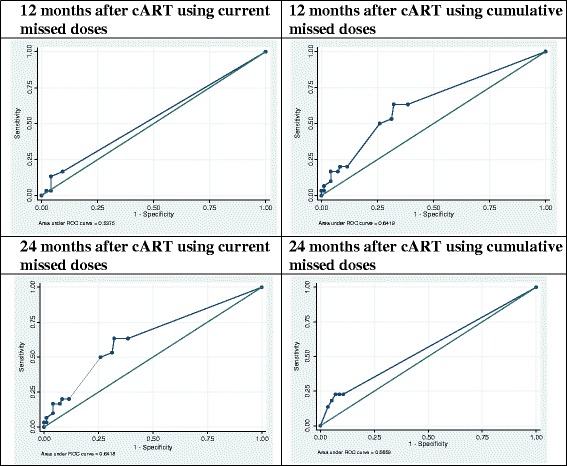
ROC curve for various reporting adherence criteria (ANRS-PEDIACAM Study, 2008-2013, Cameroon). *Legend:* cART, combined antiretroviral therapy

The area under the curve (AUC) was not significantly different to 0.5 for any missed dose criterion, except for cumulative missed doses at M12 (*p* = 0.005 for VL ≥ 1000 cp/mL) (Table 3 and Fig. 2).

### Virological failure

The proportions of infants with virological failure (defined as >1000 copies/mL) 3 and 12 months after cART initiation were respectively: 47.8 % (61/146) and 23.8 %. Twenty-four months after cART initiation, the proportion of virological failure (defined as >400 copies/mL) was 27.9 % (22/79) (Table 2).

### Performance of caregiver questionnaire about adherence

The best sensitivity was observed with the cumulative missed doses criterion as reported at M12 (63.3 %) and M24 (72.7 %). But its specificity decreased with time (to 67.7 % and 42.1 %, respectively). For all criteria used, the PPV was low (≤50 %) and NPV was high (≥75 %) at both M12 and M24. Cumulative missed doses at M12 gave the best likelihood ratios (LR + =2.0 and LR− = 0.5) (Table 3). The mixed criterion for non-adherence did not perform better at detecting virological failure.

Similar results were obtained when the analysis was restricted to the more than 70 % of the children accompanied by their mothers (Table 3).

According to both current and cumulative missed dose criteria, 11 children at M12 and 3 at M24 were classified as adherent although viral load testing indicated virological failure: they were tested for genotypic antiretroviral resistance and resistance to the currently used cART was detected in seven cases. All viruses were non-B subtypes. The most prevalent mutations in the Protease gene were I13V, K20I, M36I, H69K and L89M, and the mutations in the Reverse Transcriptase gene were T215S, M184V and Y181C.

Thirteen children identified as virological successes at M12 (VL < 1000 cp/mL) and 20 children as virological successes at M24 (VL < 400 cp/mL) were classified as non-adherent according to cumulative missed dose criteria but adherent according to current missed dose criteria.

### Factors associated with missed dose reporting

Missed dose reporting was not significantly associated with health care provider, visit time point or caregiver.

## Discussion

This study is the first to evaluate the value of a face-to-face adherence reporting questionnaire administered to caregivers for detecting virological failure 3, 12 and 24 months after initiation of cART for HIV-infected infants less than 6 months of age in an African country. As described elsewhere [13, 14, 16, 17, 19–21], we used criteria based on the number of missed doses at the time of evaluation (current) or between initiation of cART and the time of evaluation (cumulative).

The measure based on current missed doses had only low sensitivity; cumulative missed doses reported at M12 performed better for detecting virological failure (significant AUC test with p = 0.010, LR + =2.0 and LR− = 0.5). This means that the probability of classifying a child as “non-adherent” is 2 times higher if he/she is in virological failure than success. Conversely, the probability of being classified as “adherent” is 2 times higher in cases of virological success than failure.

For all criteria considered, the negative predictive values were mostly higher than 75 % but positive predictive values were mostly below 50 %. Few relevant studies report estimates of likelihood ratios and predictive values. Predictive values are more helpful than specificity and sensitivity for clinical decision-making in routine practice, but depend on the prevalence of virological failure in the population studied. Here, prevalence of virological failure was 47.8 % at M3, 23.8 % at M12 and 27.9 % at M24, which explains the high NPV. A cohort study in Uganda reported that 28.8 % (17/59) of children had viral loads ≥1000 copies/mL at M12, and this is consistent with our findings [20].

Some children were classified as non-adherentaccording to cumulative missed dose, because of poor adherence at the beginning or part way through cART although they subsequently improved and obtained virological success at M12 or M24. This led to false positive cases, erroneous detection of virological failure and consequently decreased specificity, LR+ and PPV, particularly at M24. The single measures of viral load at M12 and M24 did not allow us to examine short-term effects of adherence fluctuations on virological outcomes [14].

Other children were classified as “adherent” although they showed virological failure at M12 or M24, which would be considered false negatives, reducing the sensitivity, NPV and LR+ of caregiver adherence reporting as a test for virological failure. Among the 11 infants presenting with these “divergent” unexpected results*,* 7 had genotypic evidence of resistance to antiretrovirals in their regimens*.* The findings from this study strongly suggest that in the majority of cases, patients whose caregivers report high adherence and have virological failure have resistance mutations*.* The caregiver desire to please health care providers, as reported in previous studies [4, 12, 22–25], account for a minority of discrepant adherence-virological response findings.

Other factors which were not assessed by our study may be involved, such as concomitant disease, insufficient doses of ARV at home, drug interactions, individual differences in drug absorption and metabolism [4].

The sensitivity of the current missed dose measure for detecting virological failure was low and did not significantly vary over time during the first 2 years of cART. Reporting a non-null number of missed doses is likely to indicate that some of the doses was taken only in part, rather an exact number completely not consumed [13]. This type of measure was similarly found in a Zambian study to have low sensitivity after 24 months of cART in adults with median ART duration of 2 years [25]. According to the current missed dose measure, the proportion of non-adherent children at M12 in our cohort (10.4 %) was similar to those reported in older children in South African (13.8 %) and Ugandan (14 %) studies [14, 22]. The proportion of non-adherence at M24 in a Ugandan study of children aged 2–10 years (3.3 %) [17] was smaller than that in our study (13.9 %), possibly because the administration of medication to older children is easier.

Our study has some limitations including sample size to be analyzed at M12 and in particular at M24 being smaller than the number of children initially eligible for the study. This may have reduced the statistical power (sensitivity) for detection of virological failure. Viral load measurements were not made for some children because of death, loss to follow-up and caregiver decision to leave the health facility before blood could be drawn at the lab for plasma HIV-1 RNA tests. The ANRS 12140-PEDIACAM initially planned to follow HIV-infected infants until 2 years of age; however, many of the children who reached age 2 years (65 of 144) had not completed 2 years of cART and their viral loads were not subsequently measured. There was no association found between absence of viral load measurement at M12 or M24 and baseline characteristics.

## Conclusions

In conclusion, the adherence questionnaire administered by the health care provider to the infant’s caregiver is not a useful tool for detecting virological failure. As described for older children, its performance is poor, and its positive predictive value is too low for use in routine practice. Therefore, viral load measurement remains the best approach in routine practice to managing children under antiretrovirals and to avoid the emergence of resistance. We nevertheless report that the adherence questionnaire has a relatively high negative predictive value; adherence questionnaires and caregiver reporting may be useful in similar resource-limited settings, not to detect virological failure, but to identify virological success or resistance to antiretrovirals (in case of clinical or immunological discordance). Indeed, two or fewer missed doses reported in all trimestral questionnaires during the 12 months following the initiation of treatment is reassuring for the continuation of the treatment unchanged if the clinical and immunological status is satisfactory. Discordance suggests that it may be valuable to interview the family carefully, or propose home visits to identify any difficulties experienced with administration of medications, or to conduct genotyping tests.
